# Supplementary material for: Self-rectifying resistive memory in passive crossbar arrays
Source: Nat Commun. 2021 May 20;12:2968. doi: 10.1038/s41467-021-23180-2 (PMC8137934; doi:10.1038/s41467-021-23180-2)
Supplement: Supplementary file 1 — Supplementary Information [file 41467_2021_23180_MOESM1_ESM.docx]

Supplementary Information

Self-rectifying resistive memory in passive crossbar arrays

Kanghyeok Jeon, Jeeson Kim, Jin Joo Ryu, Seung-Jong Yoo, Choongseok Song, Min Kyu Yang, Doo Seok Jeong* and Gun Hwan Kim*

**Supplementary Fig. 1: Retention characterization at higher temperature.** The retention characteristic at higher temperature (125 °C for 2 h) to confirm the stable data non-volatility. Up to 125 °C, the data retention and memory window are maintained stably.

**Supplementary Fig. 2: Rutherford Backscattering Spectroscopy (RBS) measurement.** (a) and (b) show the RBS measurement results to identify the chemical composition of HSO^1^ and HSO^2^, respectively.

**
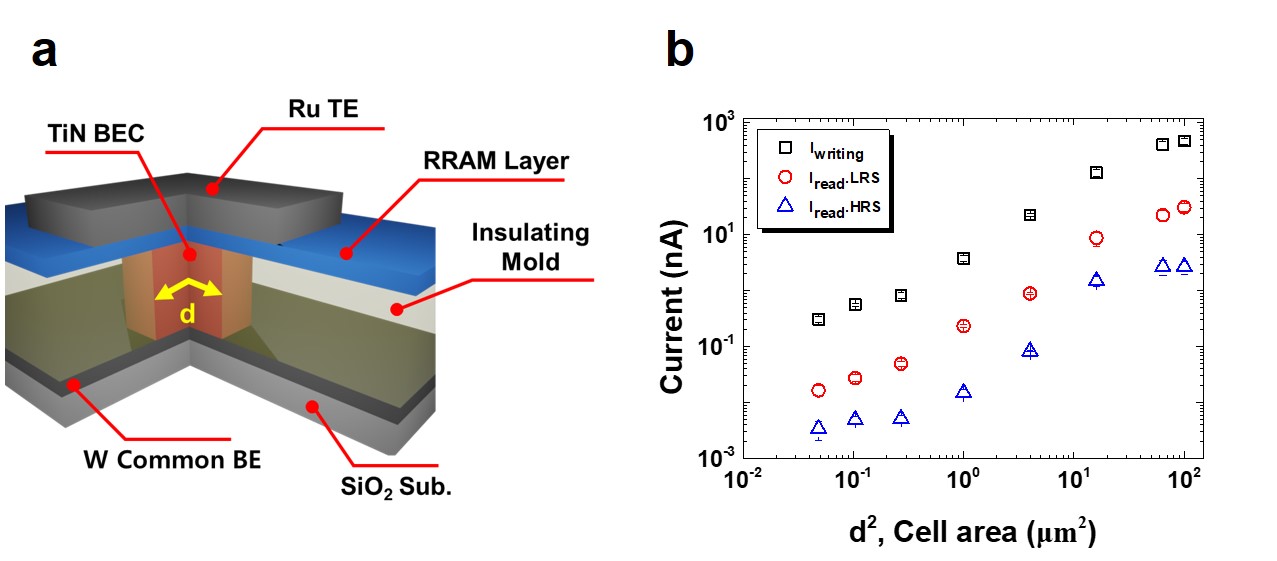
**

**Supplementary Fig. 3: Active area dependent electrical characteristic. (**a) Schematic view of SRMC device for investigating of active area dependent SRMC behavior and (b) the experimental result of current variation as a function of active area

To investigate the resistive switching mechanism of the SRMC device, the active area dependent resistance state variation was measured. Supplementary Fig. 3a shows the schematic view of the tested device structure. The various active area (0.0484–100 μm^2^) of TiN plugs (bottom electrode contact, BEC) were formed within the SiO_2_ mold for mechanical and electrical purposes. The thin film layers (HSO^1^/Al_2_O_3_/HSO^2^) for SRMC were deposited, and the Ru TE was sequentially patterned. As shown in Supplementary Fig. 3b, the current verified at 2 V of LRS and HRS increase as the active area increase, which strongly demonstrates the interface-type SRMC.

| Parameter | IL1 | HSO^1^ | | Al_2_O_3_ | HSO^2^ | | IL2 |
| --- | --- | --- | --- | --- | --- | --- | --- |
| Diffusion coefficient  *D*_Vo_ [cm^2^/s] | 2×10^-20^ | 10^-18^ [48] | | 10^-19^ [49] | 10^-18^ [48] | | -  (blocking) |
| Thickness  *d* [nm] | 0.5 | 1 | | 0.5 | 2 | | 0.5 |
| Dielectric constant $\epsilon_{r}$ | 10 | 20 | | 20 | 20 | | 10 |
| Reference state chemical potential of oxygen vacancy  $\mu_{Vo}^{0}$ [eV] | - | 0.15 | | 0.075 | 0 | | - |
| Parameter | | | | | | | |
| Band offset $\phi_{b}^{0}$(TiN/HSO^2^) [eV] | Band offset $\phi_{b}^{0}$(Ru/HSO^1^) [eV] | | Vacancy density at Ru interface | | | Vacancy hopping distance  *a*_Vo_ [nm] | |
| 0.73 | 0.84 | | 10^20^ cm^-3^ | | | 0.5 | |
| Parameter | | | | | | | |
| Electron mobility  *M*e [cm^2^/V·s] | Temperature  *T* [K] | | ESR resistance  *R*_ESR_ [Ω] | | | Device area  A [μm^2^] | |
| 0.1 | 383 | | 10 | | | 4 | |

**Supplementary Table. 1: Parameters of modeling for SRMC.**

**
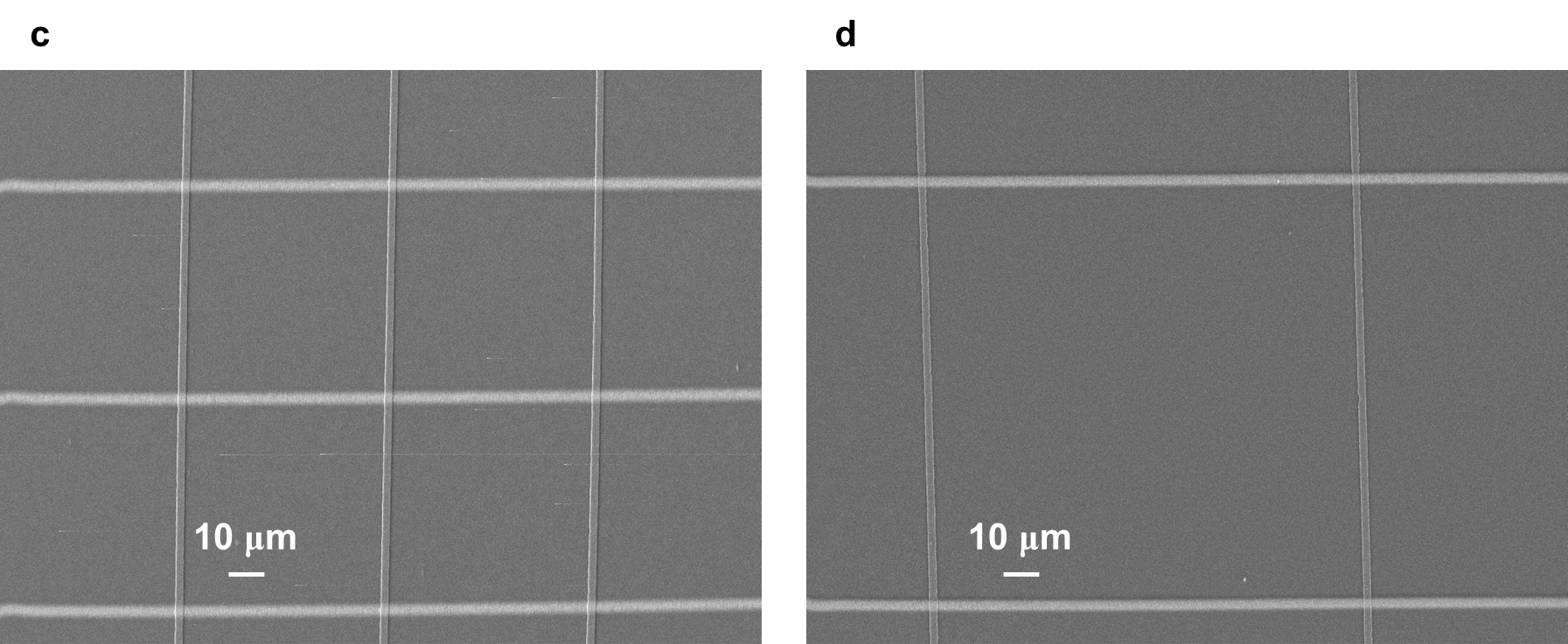
**

**Supplementary Fig. 4: Device layouts of CAs.** (a) and (b) show the device layout for CAs with 100 kb and 25 kb of integration density, respectively. (c) and (d) show the top view of scanning electron microscope image of 100 kb and 25 kb array, respectively. The electrically 4-terminal structure was adopted. The *V_op_* and *Ground* are for the signal lines of biasing and electrical ground, respectively. (for selected cell) Each *Row-* and *Column-inhibit lines* share all parallel signal lines except for the *V_op_* and *Ground*. With this CA structure the SRMC characteristic of the selected cell could be measured with the interference effect from other cells according to various bias schemes.


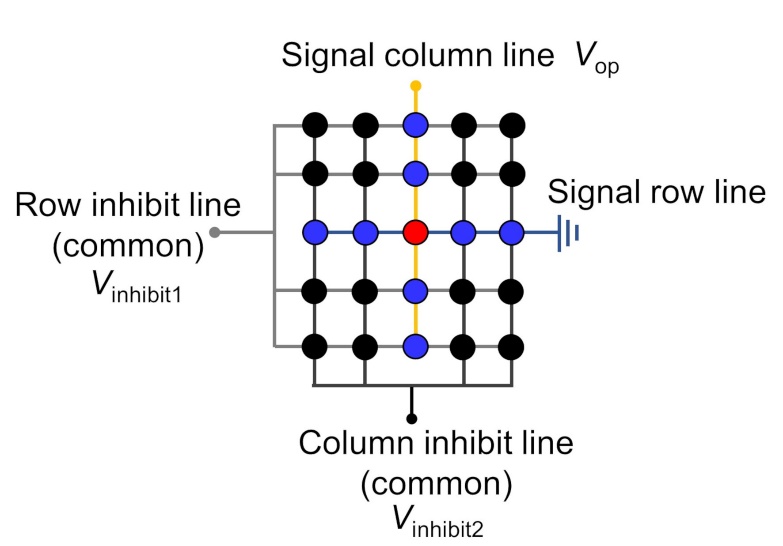


**Supplementary Fig. 5: Configuration of large CA measurement.** Configuration of a large CA that is not random-accessible.

**
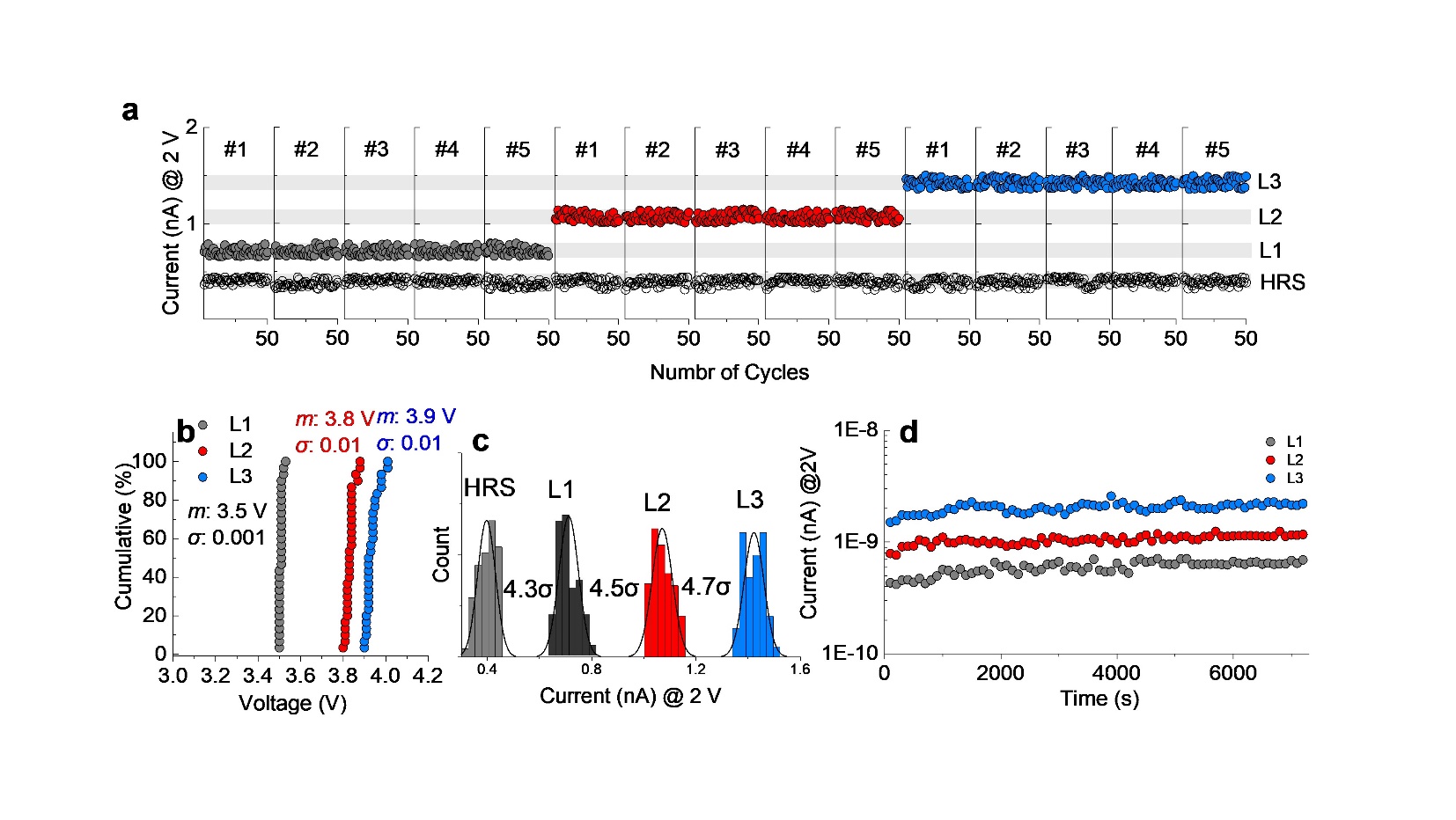
**

**Supplementary Fig. 6: Two-bit operation of SRMCs in the 30 × 30 CA.** Two-bit states programmed using the erase-and-program scheme on five SRMCs (indexed #1–#5) in the 30 × 30 CA. **b** Cumulative distribution of amplitudes of two-bit programming pulses. Average amplitude and standard deviation denoted by *m* and *σ*. **c** SOP between two-bit states. **d** Retention of two-bit states at 85 °C.


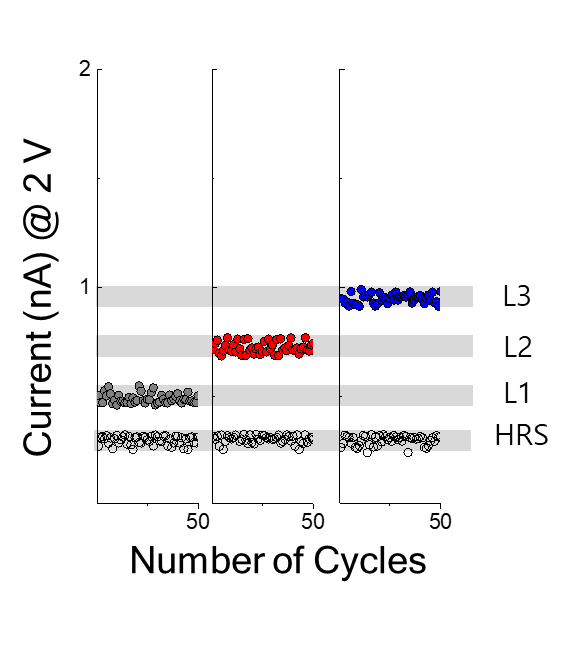


**Supplementary Fig. 7: Two-bit operation of SRMCs in the 320 × 320 CA.** Two-bit states programmed using the erase-and-program scheme on the predefined SRMC in the 320 × 320 CA.
